# Epigenome data release: a participant-centered approach to privacy protection

**DOI:** 10.1186/s13059-015-0723-0

**Published:** 2015-07-17

**Authors:** Stephanie O. M. Dyke, Warren A. Cheung, Yann Joly, Ole Ammerpohl, Pavlo Lutsik, Mark A. Rothstein, Maxime Caron, Stephan Busche, Guillaume Bourque, Lars Rönnblom, Paul Flicek, Stephan Beck, Martin Hirst, Henk Stunnenberg, Reiner Siebert, Jörn Walter, Tomi Pastinen

**Affiliations:** Centre of Genomics and Policy, Department of Human Genetics, McGill University, Montreal, QC H3A 0G1 Canada; Department of Human Genetics, McGill University and Genome Quebec Innovation Centre, Montreal, QC H3A 0G1 Canada; Institute of Human Genetics, University Hospital Schleswig-Holstein, Campus Kiel & Christian-Albrechts-University Kiel, 24105 Kiel, Germany; Saarland University, 66123 Saarbrücken, Germany; Institute for Bioethics, Health Policy and Law, University of Louisville School of Medicine, Louisville, KY 40202 USA; Department of Medical Sciences, Science for Life Laboratory, Uppsala University, SE-751 85 Uppsala, Sweden; European Molecular Biology Laboratory, European Bioinformatics Institute, Wellcome Genome Campus, Hinxton, Cambridge, CB10 1SD UK; Medical Genomics, UCL Cancer Institute, University College London, London, WC1E 6BT UK; Centre for High-Throughput Biology, University of British Columbia and Canada’s Michael Smith Genome Sciences Centre, BC Cancer Agency, Vancouver, British Columbia V5Z 4S6 Canada; Department of Molecular Biology, RIMLS, Faculty of Science, Radboud University, 6500 HB Nijmegen, The Netherlands

## Abstract

Large-scale epigenome mapping by the NIH Roadmap Epigenomics Project, the ENCODE Consortium and the International Human Epigenome Consortium (IHEC) produces genome-wide DNA methylation data at one base-pair resolution. We examine how such data can be made open-access while balancing appropriate interpretation and genomic privacy. We propose guidelines for data release that both reduce ambiguity in the interpretation of open-access data and limit immediate access to genetic variation data that are made available through controlled access.

**Electronic supplementary material:**

The online version of this article (doi:10.1186/s13059-015-0723-0) contains supplementary material, which is available to authorized users.

Sequencing-based techniques such as integrative transcriptomic measurements of gene expression and epigenomic measurements of chromatin structure are increasingly applied to the study of genome function . Open sharing of human epigenome data is of great importance to progress in the large-scale data-intensive biomedical research carried out by the International Human Epigenome Consortium (IHEC), of which we are members. Data-sharing facilitates subsequent research, enhancing reproducibility and the translation of research into new knowledge of health and disease.

Evidence suggests that genetically mediated variation within human tissues is abundant, easily mapped and shared between tissues [1]. From a genomic privacy standpoint, DNA sequence information can lead to the re-identification of research participants’ data by genetic matching — this has been referred to as ‘attribute disclosure attacks using DNA’ (ADAD) [2]. Here, we discuss the current practices and privacy protections currently available for the release of genomic and related data. We quantify the extent to which identifying DNA sequence information confounds anonymization using the example of methylation data, and conduct an ethical-legal analysis of the issues raised with respect to the privacy and autonomy of research participants. Finally, we propose open-access data-release policies to address these issues.

De-identification of data by removing direct identifiers (such as participants’ name, date of birth, social insurance numbers and facial images) is widely used for shared research data. In North America, anonymization implies that the de-identified data are no longer linked to any identifiers. By contrast, coding refers to an alphanumeric ‘code’ that links de-identified data to identifiers. In this analysis, we draw a distinction between the re-identificatin of data — its attribution to an individual by matching identified (named) genetic information to anonymized data — and the potential to link two anonymized datasets. Absolute anonymization of even small amounts of DNA sequence information can be impossible given the extent to which DNA sequence is unique to individuals [3, 4], but epigenomic data lend themselves more readily to anonymization.

When there is a reasonable risk that data can be re-identified, or there are limitations on the use of the data in different types of analyses, another strategy to enable the data to be shared is to control access to it. ‘Controlled access’ (‘managed access’) has generally been applied to data types that provide extensive DNA sequence information from an individual. Researchers must apply for access to such datasets and be approved by a ‘Data Access Committee’ (DAC). The ability to re-identify and misuse research data is considered less likely when the data are shared under controlled access arrangements that involve a review of applicants’ credentials, a review of their research plans, verification that the proposed research has been approved by an ethics committee or that a waiver has been obtained, and the signing of a contract referred to as a Data Access Agreement that forbids (amongst other things) the re-identification of data. DACs can also provide some degree of post-authorization oversight of data use [5]. These measures can, to varying degrees, limit data access and analysis, so they have been perceived by some members of the research community as hindering ‘crowd-sourcing’ or collaborative analysis of publically funded genomic datasets [6]. Other concerns include delays that result from the controlled access process and its lack of transparency [7].

Numerous security strategies can increase the level of protection of data (for example, firewalls or encryption) or enhance privacy (for example, iDASH [8] and Bio-PIN [9]). Typically though, data security measures serve to reinforce controlled access distribution and do not address its main limitations: restricting acceptable data use and aggregation. An emerging approach to providing broad access to data while protecting the privacy interests of research participants is that of data ‘safe havens’ — protected IT environments for pooling data (such as DataSHIELD [10]). The strengths of this approach are that it aims to reduce the risks of distributing large amounts of data to individual researchers and decreases reliance on contracts and other legal protections that are neither fail-proof nor evenly provided internationally, and which can be difficult to enforce.

Following the model of the National Institute of Health (NIH) Roadmap Epigenomics project, an IHEC partner, processed IHEC epigenomes are publically accessible in appropriate data archives, track hubs or similar summary data formats. Associated raw sequence data and metadata information are also shared, either through open-access or controlled-access mechanisms. Similarly, The Cancer Genome Atlas (TCGA) provides publically accessible ‘Level 3’ summarized methylation calls, whereas controlled access to ‘Level 1’ and ‘Level 2’ data restricts the availability of raw sequence and mutation calls [11]. Open-access data, which are freely available for anyone to use, typically include intensities of signal (such as gene expression or DNA–protein interaction) or levels of methylated cytosine. Such summary data do not report genetic variation directly, and their release reflect the strategies developed for the open-access release of array-based gene expression data by the National Centre for Biotechnology Information (NCBI) Gene Expression Omnibus (GEO) or the European Bioinformatics Institute (EMBL-EBI) ArrayExpress (AE) databases. Users must rely on the data submitter for appropriate processing of data, potentially leading to biological misinterpretation.

DNA methylation data are an example of a form of epigenetic information that can lead to misinterpreted results because of the presence of genetic variants, given its reliance on CpG (cytosine-phosphate-guanine) dinucleotide contexts (CpGs) as the unit of information. Other components of epigenome mapping data (such as DNase hypersensitivity sites or chromatin marks [12–14]) also show evidence of genetic governance, but the density of these traits and how they are shared across tissues has only been studied in smaller datasets. Bisulfite conversion causes unmethylated cytosines to be converted to uracil, allowing methylated and unmethylated cytosines to be distinguished. Whole-genome bisulfite sequencing (WGBS) is a high-throughput, genome-wide DNA methylation interrogation technique that reports methylated and unmethylated cytosines at CpG sites within a reference genome.

WGBS is biased at the start and end of reads because it includes unmethylated cytosines that are added during overhang repair and 5′ underconversion from adapter re-annealing [15, 16]. It also confounds methylated cytosines and hydroxymethylcytosines, which are of particular importance in certain cell types (for example, in the nervous system) [17, 18]. We focus on genetic confounders: WGBS additively measures the frequency of cytosines in CpH (cytosine-phosphate-(non-guanine nucleotide)) contexts, as well as thymine polymorphisms.

## Case study: genetic information in methylation data

**Fig. 1:**
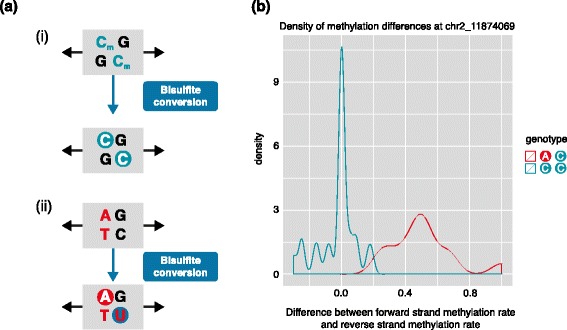
Genotypic differences in forward and reverse strand methylation. **a** (i) On reads from both strands of the wild-type C allele, the methylated C usually remains as C after bisulfite conversion, and is counted as methylated. This results in a mean difference of methylation between the strands of 0. (ii) For the allele where the methylated C is replaced by A, reads on the forward strand have the A at the CpG site and are not counted, whereas the reads on the reverse strand have the C bisulfite-converted to U and are counted as unmethylated. This results in a mixture of methylated and unmethylated reads on the reverse strand, whereas there are only methylated reads on the forward strands. **b** Heterozygotes that have A and C alleles (*red*) are compared with homozygotes that have two copies of the C allele (*turquoise*). We see negligible difference in methylation rate between forward and reverse strands in the 26 homozygous individuals, but an average of around 50 % more methylation on the forward versus the reverse strand in the 13 heterozygous individuals

Strand-specific WGBS measures CpG methylation for the forward and reverse strands independently, but both strands usually have concordant methylation rates. Nevertheless, when the cytosine of the CpG is mutated to adenine or guanine on the forward strand, asymmetric methylation rates are measured (Fig. 1). When the cytosine is mutated to thymine, all reads are counted, but forward reads that contain the thymine mutation are miscounted as bisulfite-converted unmethylated cytosines, and reverse reads measure CpH methylation at the mutated site. In both cases, the polymorphism can be detected by the base-paired genetic variation in reverse reads [19, 20] or externally by direct genome sequencing or genotyping arrays.

**Fig. 2:**
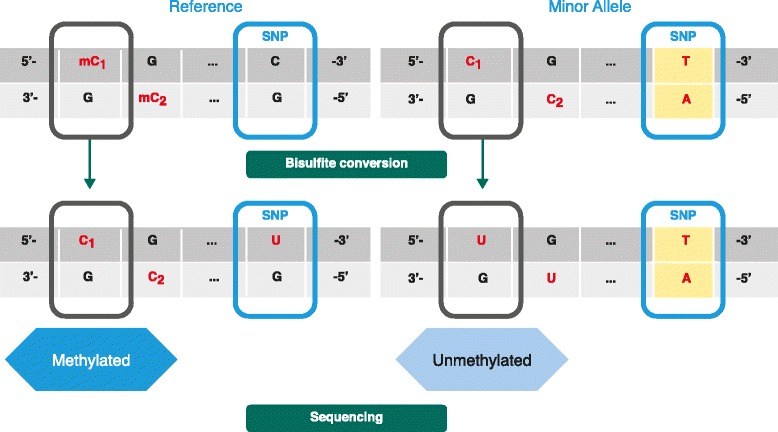
Example in which methylation is indirectly affected by a SNP. The CpG site is normally methylated (*left*) when the genomic sequence at a downstream SNP position is a C. When the downstream SNP is mutated to a T, the CpG site is affected and becomes unmethylated, allowing the conversion of the cytosine residues at the CpG site to uracil (right)

**Fig. 3:**
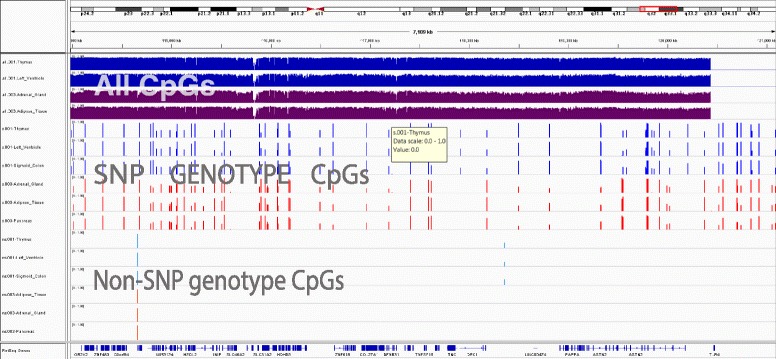
Example CpG sites showing correlation to genotype on chr9:115,000–120,000. *Blue* or *turquoise* bars show methylation for the individual STL0001, *purple* or *red* bars show methylation for the individual STL003. Each track shows DNA methylation patterns in a different tissue sample from one of the two individuals. Overall, DNA methylation patterns in the two individuals appear to be similar (*top four tracks*), but we can see a distinct, individual-specific pattern of methylation at CpGs overlapping SNPs (*shaded box*, *middle tracks*) and, much rarer, at CpGs not overlapping SNPs (*shaded box*, *bottom tracks*)

We identified genomic CpGs from WGBS in which the measured methylation rate is due to genetic rather than epigenetic variation and is independent of tissue type (Fig. 2). We did this by filtering for CpGs that have a static methylation rate in all tissues from the same individual in the NIH RoadMap Epigenomics (Roadmap) [21] WGBS samples (Additional file 1: Table S1) but which vary between individuals. A total of 5.9 million candidate CpGs were identified from a pool of 24 million well-measured CpGs present in most of the Roadmap samples, extrapolating to potentially 7.4 million candidates among the 30 million CpGs genome-wide (assuming that CpGs that are unassessed by lower sequence coverage have similar distribution). When 3.6 million CpGs were evaluated using McGill Epigenome Mapping Centre (EMC) [22] WGBS and single nucleotide polymorphism (SNP) array data, 443,636 CpGs showed correlation (R > 0.5, *p* < 0.05) with the presence of an array-genotyped SNP within 10 kb. Of these, 354,710 (80 %) CpGs directly overlapped a known SNP in dbSNP137 (Fig. 3). Of the genotype-correlated CpGs, 67,913 showed high (>98 %) predictive accuracy, with 53,294 CpGs (78 %) directly overlapping a known SNP. Of the highly predictive genotype-correlated CpGs, 39,000 remained after the removal of sites where forward and reverse strand methylation rates from WGBS are discordant, another criterion used to filter the genetic variation.

Public WGBS datasets therefore contain thousands of genetic variants, predominantly known common variants, that disrupt CpGs. Other sites that show high variability among individuals, but not tissues, may be subject to indirect genetic effects or may contain rare variants. We validated the Roadmap/EMC-identified highly predictive genotype-correlated CpGs using independent methylation and genotype sequencing data from adipose tissue [23]; only CpGs overlapping a known dbSNP137 SNP remained correlated in validation (0/24 CpG sites not on a known SNP remained correlated to the genotyped SNPs in validation). While thousands of CpG-disrupting SNPs reporting CpG methylation were found in public databases, no true ubiquitous ‘epigenotypes’ at actual CpGs were validated. Uncalled genetic variants that disrupt the CpG context were highly enriched among sites that were ‘differentially methylated’ between individuals, but had low inter-tissue variation within individuals. Tissue-specific sites that are ‘differentially methylated’ in different individuals are also probably enriched for genetic variation, but intra-tissue indirect genetic influences will be substantial [1].

**Fig. 4:**
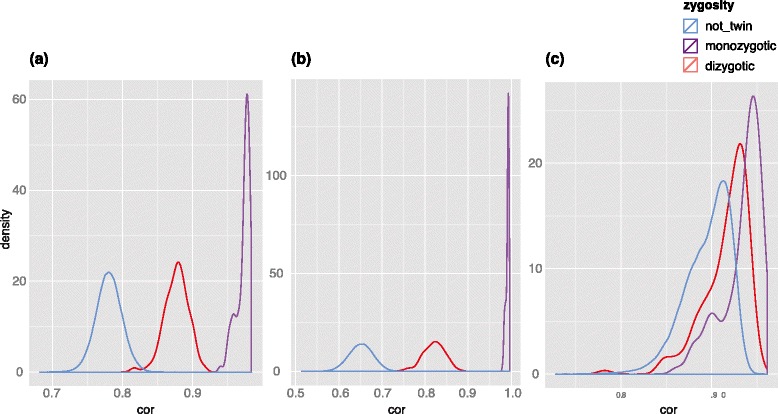
Density of pairwise CpG methylation correlation between adipose tissue samples at selected CpGs. Pairwise correlation was calculated between all possible pairs of TwinsUK adipose tissue samples. **a** All of the selected 1306 genotype-correlated CpGs on the 450 K array. **b** One or more SNPs or mapping multiple sites are overlapped by 699 probes. **c** For 607 probes, there is no SNP in the probe-binding region. Correlations at these sites between monozygotic twins is extremely high (*green*), whereas dizygotic twins are correlated to a lesser degree (*red*) and unrelated individuals have markedly lower correlation (*blue*)

Other methylation interrogating techniques also expose genetic information. The Illumina Infinium HumanMethylation 450 K BeadChip Array (450 K) provides genome-wide microarray interrogation of 485,577 CpG targets. We identified probes from public domain 450 K data that had a static methylation rate in all tissues from the same individual but which had variable methylation rates between individuals. After excluding all 65 SNP-targeting ‘rs’ probes, 1306 ‘cg’ probes (Additional file 2: Table S2) matched leukemia cancer and normal cells by genotype [24]. When validated in adipose tissue [25], these probes showed extremely high correlation in monozygotic twins compared with that in dizygotic twins and unrelated individuals (Fig. 4).

## Removing direct genetic variation

The strand-specific WGBS approach allows unequivocal distinction between genetic and epigenetic variation through direct sequencing of base-paired nucleotides at the same position as the variation on the opposing strand. Using Bis-SNP [19], we identified the genotype of reference CpG sites from normal purified blood WGBS datasets de novo (without dbSNP information), validating against heterozygotes detected in genotyping arrays. We identified 66.5 % of arrayed variants at CpGs, reducing the fraction of CpG sites that contained variants from 11 % to 3.7 % of the genotyped CpGs. Of the genotyped positions overlapping CpGs, 0.029 % were incorrectly called (false positive SNP or incorrect variant called). Low coverage (<10 reads) contributed to the vast majority of the mislabeling.

Using SNP frequencies from dbSNP137, a median of 95 % of covered reference CpG positions in the WGBS data were retained after removing detected SNPs and unclear cytosine contexts. Detection of variants at genotyped CpGs was increased to 75 %, and erroneous SNP calls were reduced to 0.024 %. When focusing on high-coverage CpG sites (with a minimum of 15× coverage), we identified 0.4–1.5 % (median 1.3 %) of high-coverage CpGs per sample as having SNPs (samples had 1 million to 20 million high-coverage CpGs, median 5.7 million).

**Fig. 5:**
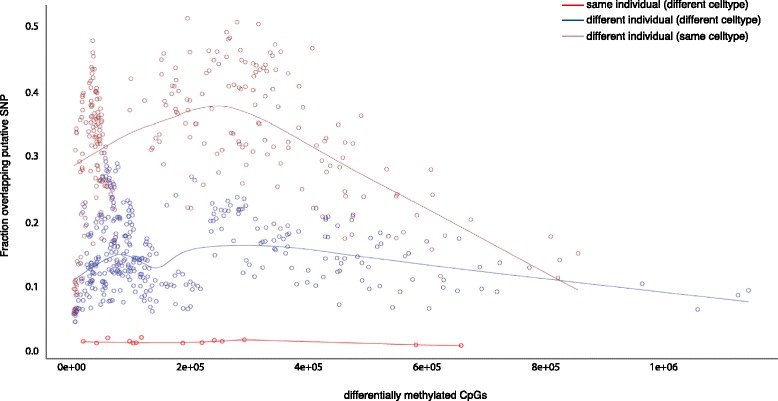
Fraction of differentially methylated CpGs that overlap Bis-SNP observed SNP position compared with coverage. We observed relatively low numbers of differentially methylated CpGs overlapping SNPs when comparing cell types of the same individual (*red*), and high numbers overlapping SNPs when comparing the same cell type from different individuals (*brown*). An intermediate number overlap SNPs when both cell type and genotype are varied

We next examined differentially methylated CpGs (methylation rate difference >30 %) between pairs of samples. Overall, between 1 % and 50 % of the differentially methylated cytosines (median 20 %) were identified as overlapping sequence variants in one or both samples (Fig. 5). When comparing the same blood cell type between different individuals, an extremely large fraction (up to 50 %) of differentially methylated CpGs were due to SNPs (median = 33 %). By contrast, samples from different cell types of the same individual (16 pairs in total from 7 individuals) showed a median of 1.5 % overlap with SNP calls, indicating that differential methylation at heterozygous sites is rare. Varying both tissue and genotype, SNPs had an overall intermediate contribution to the differential methylation (median 14 %) at CpG sites, indicating that while CpGs that have true differential methylation were detected (above the intra-tissue rate), genetic variation at the CpG site remained a substantial influencing factor.

## Vulnerability of metadata

There remains a very small risk of re-identification of research participants by matching their identified named genomic information to data from a study participant. We therefore consider the consequences of potential re-identification of associated clinical/healthcare information and other lifestyle or demographic information, which may be studied and available from metadata and study parameters. Some of these metadata may also increase the likelihood of re-identification of the dataset.

Epigenome mapping projects include samples from a number of population cohorts with varying health conditions, including rare diseases. It is clear that the epigenome is impacted by disease state; therefore, some categorization of the health status of the donor may be necessary depending on the tissue studied. The use of controlled vocabulary with disease ontologies (such as the NCI Metathesaurus used by IHEC) allows for this information to be reported in a standardized manner, which reduces the risk of inadvertent disclosure of more detailed health information if a dataset were to be re-identified. Nevertheless, some medical information does not correspond neatly to existing ontology terms and it may be necessary to allow for additional ‘free-form’ text relating to disease and donor health status.

For individuals with a rare disease or other rare phenotype, disease or donor health status information could potentially increase the risk of re-identification of epigenomic data in the same way as seemingly innocuous ‘demographic’ information. For example, full date of birth and place of residence have been shown to enable re-identification of healthcare data in some circumstances [26]. Information on rare disease status can increase the risk of re-identification not only because rare diseases are rare, but also because the disease often presents outwardly visible characteristics that could link a whole dataset more rapidly to an individual. Furthermore, some rare diseases imply potential carrier status for relatives and the disease may also be associated with potentially stigmatizing information. For example, bilateral striopallidodentate calcinosis, with fewer than 200 known cases and for which the genetic basis is not fully understood (familial and sporadic forms, genes unknown) may cause personality changes and dementia [27]. Mental health information is generally considered to be stigmatizing and it is often provided special protection by law [28]. Severe conditions such as this are, however, unlikely to be kept private once symptomatic, so the main risk is the increased likelihood of re-identification of other information in the dataset.

Rare disease information may also reveal an individual’s likely ancestry or geographical location. For example, Tay-Sachs disease has a higher prevalence in individuals of Ashkenazi Jewish descent [29], and Leigh syndrome in the Saguenay-Lac-Saint-Jean region of Quebec [30]. In some cases, such associations may result in a loss of privacy. Furthermore, the experience of projects in which rare disease genetics data have been shared indicates that patients and their families are willing to accept voluntarily the risks associated with potential re-identification if they have been explained to them. While this acceptance of risk may not be greater than in other research circumstances, it can be presumed that there are greater expectations of benefits from involvement in rare disease research. We propose points to consider for assessing the risk of sharing rare disease information in open-access data sets (Table 1). These relate to the potential for re-identification, the privacy and sensitivity of rare disease data, and research participants’ consent.

**Table 1:** Points to consider when sharing rare disease information

Points to consider	
1	Is the place of residence provided (even indirectly, for example, in the project name)?
2	Is the rare disease outwardly visible?
3	How rare is the disease?
4	Does the rare disease provide information about the likely geographical location of individuals?
5	Does the rare disease provide information about ethnicity that may be considered potentially stigmatizing?
6	Was the participant aware of the potential risks of data re-identification?

While it is very difficult to quantify the likelihood of re-identification in these cases, a ‘rarity’ threshold for point 3, for example, could be considered that would be relative to the availability of information on place of residence and the visibility of the disease (points 1 and 2). If the answer to point 4 or 5 is yes, we recommend holding rare disease information in ‘controlled access’ while clearly indicating its availability.

Most current epigenome mapping projects focus on the characteristics of human cell types or tissues and de-identification is the norm. Nevertheless, datasets commonly include two other important categories of metadata — donor age and ethnicity — which impact interpretation of the data and are therefore important to share as openly as possible [31, 32]. The risk of re-identification of anonymized datasets from ‘demographic’ metadata requires project-specific consideration, depending mainly on other sources of available information and on the group sizes of a given demographic [26]. Standards, such as the US Health Insurance Portability and Accountability Act (HIPAA) Privacy Rule, significantly decrease re-identification risk (for example, by using age, not date of birth, with a category for ages over 90 years) [33].

For ethnicity, the risk mainly applies to minority groups, with the re-identification risk varying (similar to that for rare disease metadata). Ethnic origin or ethnicity is included as a surrogate marker for genetic similarity or relatedness in order to improve the quality of research results in terms of their significance generally and for individuals [34–36]. This metadata use creates difficulties with respect to adopting publically acceptable group designations [37]. Given the diversity of approaches for recording ethnicity (or not) in different parts of the world, and the benefits of standardizing descriptors in research, consulting local census categories and assigning a limited set of choices based on the populations studied would help in addressing social and political issues that might affect research participants [38]. However, populations requiring special attention, such as small ethnic groups that may be more prone to the risks of re-identification, need to be identified as such if their data are to be shared with extra protections. This can lead to a quandary as census categories may purposely avoid asking for this information. We suggest reviewing lists of proposed descriptors for sample populations, and, if possible, providing preset lists to select from that are based on locally acceptable designations such as those of national census categories. For small or vulnerable populations, the determination of which will also usually depend on local context, we also suggest moving this information (and potentially other data from these individuals) to the ‘controlled access’ portion of the data.

## Mitigating risk for data release

Anonymized genome-wide DNA sequence information that is contained within public repositories can be linked to individual participants [2]. The main reason this has not prevented its public release in some circumstances (for example, with appropriate consent and following an assessment of the sensitivity and identifiability of associated metadata) is that, in the vast majority of cases, to do so would require access to an individual’s identified genetic data from another source, in which case the information, health-related or otherwise, that it contains would probably not be protected. Anonymized genome-wide genetic data can also sometimes be re-identified *by* other routes, such as through surname inference for well-documented collections [39]. Furthermore, for functional genomic data (such as RNA-expression profiles), considerable efforts would be required to match datasets by tissue of origin and processing techniques. This has been studied for gene expression arrays using pre-existing knowledge of genetic variation that impacts gene expression differences in populations [40] and is a much more complex route to a privacy breach [2].

Open-access DNA methylome data contains DNA-sequence information that could potentially be used as re-identifying information through genetic matching. However, the majority of genotype-resolving CpGs in WGBS data directly overlap known SNPs, representing other sequence contexts misleadingly released in CpG-methylation tracks. The CpGs disrupted directly by SNPs that are currently present in open-access epigenome data resources can be efficiently removed from high-coverage data by pre-filtering prior to release using existing algorithms or genotyping resources, with minimal loss of ‘true epigenetic’ information. Over 75 % of the disrupted CpGs could be eliminated with nearly 0 % erroneous calls, affecting only 1.5 % of the methylome. The genotypically resolved raw datasets would still allow interrogation of these disrupted CpGs, and in cases such as cancer genomes, somatic mutations could be reported while keeping germline mutations under controlled access (as in the TCGA policy [11]). Unfortunately, filtering cannot be used as effectively for all data types, including that generated by non-stranded bisulfite-sequencing methods (such as post-bisulfite adaptor tagging (PBAT) [41]) and methylation array data. Nevertheless, the effects of common genetic variation could still be reduced by masking sites (CpGs or probes) that have common SNPs [42, 43]. Methylation data with direct genotype variation removed would have, in our view, very low re-identification risk, probably in the same order as that for functional genomic data. For summary-level open-access data (where the user cannot reprocess the reads), such steps should precede deposition to public archives or availability in public track hubs by data producers. Patterns of data omission resulting from variants at CpGs, the presence of undetected genetic variation, and the proven existence of strong indirect (non-CpG disrupting) genetic effects on methylation within the same tissue [1] all indicate that residual genetic information will remain within methylome profiles. We have therefore also proposed additional measures to mitigate the impact of this very remote potential re-identification risk because we see great value in openly sharing the associated health and disease information and information on age and ethnicity.

Generally speaking, the greater the likelihood of re-identification and the greater the possibility that harm may occur as a result of re-identification, the greater the precautions and safeguards ought to be. For health-related and other private information, it would not be safe to assume that individuals would not generally feel distressed and would not suffer from stigma, if not discrimination, if this information were to become widely available. The ‘reasonableness standard’ determines that only information that can reasonably be expected to identify an individual is generally considered personal or protected by privacy laws and is included in many laws and conventions addressing data protection [44]. Following this standard, our position is based on careful evaluation of the reasonable likelihood that the data might lead to re-identification of participants. A similar approach has been taken in other large-scale data sharing collaborations such as the International Cancer Genome Consortium [45]. Furthermore, the level of privacy we feel we should strive for is one at which both the likelihood of re-identification and any potential resulting harm are very low. This level of risk is justified in light of the public benefits of research, better understanding of health and disease, and better preventative, diagnostic, prognostic and treatment strategies that may result from epigenetic research. Our strategy relies on responsible data preparation and can benefit from additional ‘Points to Consider’, such as those proposed in Table 1, for assessing rare disease information.

Although documented incidents of discrimination or stigmatization on the basis of genetic information are largely limited to highly hereditary Mendelian disorders, these rare incidents have generated substantial media coverage and significant public concern [46, 47]. Several studies demonstrate that anxiety over genetic discrimination deters people from participating in promising research projects and even from undertaking clinically relevant genetic testing, even when anti-discrimination legislation has been in place for many years [48–51]. Misperception could be attenuated by providing more accessible information on privacy and anti-discrimination protections and their limitations, and a more balanced account of occurrences of genetic discrimination. Individuals might also be willing to accept the low risk of re-identification if the risks and benefits of the research are carefully explained and researchers pledge to protect the confidentiality of information to the extent possible. Information about data sharing and its risks ought to be provided during the consent process, as even consent to the broad research use of data may not be understood by participants as also implying consent to the widespread international sharing of data. This presents challenges as the risks or method of data sharing may not be known in advance. Representations of absolute protection should be avoided. Participants should also be informed that the sharing of health and other information via social media and other internet platforms may allow them to be matched to their anonymized research data. Such a patient/participant-centered approach would be respectful of participant autonomy and dignity, focusing on education and transparency, and not promising unrealistic levels of protection. The Personal Genome Project (PGP) pioneered a route for openly sharing integrated genomic, environmental and medical or trait data [52] in 2005, which was subsequently implemented in four countries (USA, Canada, UK and Austria). PGP successfully addressed many issues using an innovative open consent protocol [53]. Despite the explicit risk of re-identification, only 3.8 % of participants have withdrawn from the PGP over the past 10 years [54], suggesting high levels of participant acceptance and low levels of adverse risk from openly shared data.

Numerous regional and national laws have been enacted to protect individuals from undesired use of their medical and genetic information, particularly from genetic discrimination in insurance and employment [55]. Nevertheless, it is currently unclear whether genetic discrimination legislation would apply to all kinds of epigenetic data because of the definitions of genetic data used in such legislation [56, 57]. For example, the US Genetic Information Nondiscrimination Act, 2008 (GINA) probably would not apply to epigenetic information since under this law the definition of a genetic test is limited to ‘an analysis of human DNA, RNA, chromosomes, proteins, or metabolites, that detects genotypes, mutations or chromosomal changes’ [58]. The German law ‘Gendiagnostikgesetz’ presents a similar situation as it defines in its §3 a genetic test as a directed test to diagnose the ‘genetic characteristics’ of a person. ‘Genetic characteristics’ are defined as ‘inherited or in between conception and birth acquired, human-derived genetic information’. In the US, the enactment of the Affordable Care Act of 2010 provides important protections against genetic discrimination in health insurance because it prohibits the denial of coverage or other adverse treatment on the basis of any preexisting health conditions or health information. Thus, this law goes beyond GINA (which only applies to asymptomatic individuals) in ensuring nondiscrimination against affected individuals in health insurance coverage. In addition, requirements for ethics review of research provide additional protection in many jurisdictions.

More robust privacy and anti-discrimination laws may be needed at the national level to efficiently address epigenetic discrimination without unduly restricting the flow of research data. However, these concerns reach beyond the context of ‘OMICS’ research. Society may have to re-conceptualize and contextualize medical confidentiality and personal privacy so that they remain relevant in the context of information technology developments and the sharing of health information through social media and the World Wide Web [59]. As demonstrated by PGP [54] and advocated by the Global Alliance for Genomics and Health, we believe it is possible to reconcile privacy protection and the protection of public benefits from scientific research that uses personal information by carefully examining the risks and using tailored data-release strategies.

Epigenomic data may also convey health-related and environmental information directly (for example, history of cigarette smoking). Discussion of these issues has been initiated [56, 60], but beyond the known impacts of smoking, alcohol consumption, chronological age and certain diseases (predominantly cancers), which are often known at sampling, epigenetic signatures for environmental exposures or disease risks have not matured sufficiently to allow assessment of their impact on data-sharing practices.

Removal of direct genotype information in methylome analyses mitigates substantial re-identification risks. Confident re-identification on the basis of the remaining methylome and other open-access epigenomics data would probably require considerable efforts. While absolute privacy cannot be guaranteed with high-throughput genomic data, we have outlined a consistent approach that limits the risks associated with open-access metadata release, aiming to allow categorization of data (for example, epigenome from normal or diseased tissue) rather than performing in-depth phenotypic correlations. Ideally, solutions that provide the benefits of open-access sharing while protecting the interests of research participants will be developed. Simultaneously, efforts to improve controlled-access mechanisms and processes for granting informed consent should be pursued. These include developing standard consent information materials and data-access agreements, and streamlining and further simplifying processes for the approval of data access.

## Methods

### CpG site analysis from Roadmap Epigenomics WGBS data

We tested CpG sites reported in the NIH Roadmap Epigenomics datasets in the following manner. To assess sites for intra-individual variation, we considered only sites with measurements in at least three samples from the same individual, and we computed the standard deviation of the methylation at the interrogated site. We required over half of the individuals (three out of the five) to have a standard deviation less than 0.07 at this site (bottom 70 % in a test of 100,000 CpG sites). We filtered for a minimum level of inter-individual variability by requiring the range of the methylation among the samples to be at least 15 % (top 35 % in a test of 100,000 CpG sites).

### Internal assessment of genotype-methylation correlation

Genotypes for the samples were obtained using Illumina 2.5 M and 5 M genotyping arrays. For each CpG site, we correlated the methylation at this site against all SNPs within 10 kb. We modeled a linear relationship between the genotype at the SNP site and the methylation rate at the CpG site. This views each allele for the SNP as having an associated methylation rate for the CpG site, and the overall methylation rate at the CpG site as being the average of the methylation rates of the SNP alleles present in the individual. For each CpG-genotype pair, we use the fitted slope and intercept across all available samples to extrapolate the best-fit mean methylation rate for each of the three genotypes. To predict the genotype for a given methylation level, we selected the genotype with methylation rate closest to the observed methylation level.

### Determination of genotype from WGBS and detection of mislabeled epigenetic variation

Bis-SNP 0.82.2 [19] was applied to the aligned and filtered reads of the purified blood samples to call SNPs directly from the strand-specific sequencing data. We limited our analysis to samples with at least 10× average read coverage (24 samples with read coverage from 12× to 22×, interrogating an average of 254,000 sites per sample). We first applied Bis-SNP without providing any prior variation information from dbSNP, evaluating all sites under the worst-case assumption of rare SNPs with no prior information. Genotype of CpG-context-altering heterozygous SNPs were determined using the Illumina 2.5 M genotyping array. Genotypes extracted using Bis-SNP without prior dbSNP frequency were compared against genotyped reference CpG sites to determine the ability to detect true heterozygous mutations as well as the rate of CpGs that were falsely identified as mutated.

We subsequently investigated the prevalence of sequence variation in methylation data by running Bis-SNP using the SNP frequency information from dbSNP137, and by examining sites with substantial read coverage (≥15×) and large differences in methylation between samples (>30 %).

### Roadmap Epigenomics WGBS data

Processed graphs of methylation proportions aligned to hg19 from Roadmap Epigenomics WGBS datasets were downloaded from the NCBI GEO repository [61]. We considered samples when multiple tissues were available from the same individual, a total of 49 tissue samples across five individuals (Additional file 1: Table S1). Samples were processed for bisulfite-converted methylation sequencing as described by Lister et al. [62]. CpG sites that had at least four reads (combining reads on both strands) were reported.

### McGill epigenome mapping centre datasets

We assessed the correlation between methylation and genotypes in seven projects spanning tissues from naïve T cells (11 samples), cortical and trabecular bone (3 samples), muscle (7 samples), purified blood (29 T-cell, 20 monocyte and 7 B-cell samples) and whole peripheral blood (6 samples), crushed bone (3 samples), and adipose tissue (8 samples) (97 samples in total). Sequencing data are available through the McGill Epigenomics Mapping Portal [22]. Raw data are available through EGA under the study “McGill Epigenomics Mapping Centre” [EGA: EGAS00001000995].

We used the subset of the purified blood samples obtained from 28 normal Swedish individuals to evaluate genetic variation that had been mislabeled as epigenetic differences. A total of 37 samples were analyzed from the three purified blood cell populations (CD14- CD4+ T-cell samples, CD14+ monocyte samples and CD19+ B-cell samples).

### DNA extraction

Genomic DNA (gDNA) was isolated using the NORGEN purification kit (Norgen Biotek Corporation, Canada) according to the manufacturer’s protocol. All quantifications were carried out using Quant-iT PicoGreen (Life Technologies, Burlington, ON, Canada).

### Whole-genome shotgun bisulfite sequencing

WGBS gDNA library preparations were carried out using the TruSeq DNA Sample Prep Kit v2 (Illumina) with an added bisulfite conversion step. gDNA (1–3 μg) spiked with 0.1 % (w/w) unmethylated λ DNA (Promega, Madison, WI, USA) was fragmented to 300–400 bp peak size using the focused-ultrasonicator E210 (Covaris, Woburn, MA, USA) to generate double-stranded DNA with 3′ or 5′ overhangs. Fragment size distribution was controlled on a Bioanalyzer DNA 1000 Chip (Agilent, Mississauga, ON, Canada). End repair, sample purification with AMPure beads (Beckman Coulter, Mississauga, ON, Canada), adenylation of 3′ ends, and adaptor ligation was carried out as per Illumina’s recommendations. The ligation product was cleaned up by one AMPure purification step, the purified DNA then analyzed on a Bioanalyzer High Sensitivity DNA Chip (Agilent), and quantified by PicoGreen before undergoing bisulfite conversion using the Epitect Fast DNA Bisulfite Kit (Qiagen, Toronto, ON, Canada) according to the manufacturer’s protocol. Bisulfite-converted DNA was quantified using OliGreen (Life Technologies), and based on quantity amplified by four to six cycles of PCR using the Hifi Uracil + DNA polymerase (Kapa Biosystems, Woburn, MA, USA) according to the manufacturer’s protocol. Amplified libraries were validated and quantified on Bioanalyzer High Sensitivity DNA Chips and underwent 100 bp paired-end sequencing on Illumina HiSeq2000 or HiSeq2500 systems.

Generated reads were aligned to the bisulfite-converted reference genome using the Burrows-Wheeler Alignment tool (BWA). A number of reads were removed as described by Johnson et al. [63]: (i) clonal reads, (ii) reads with low mapping quality score (<20), (iii) reads with more than 2 % mismatch to converted reference over the alignment length, (iv) reads mapping on the forward and reverse strand of the bisulfite converted genome, (v) read pairs not mapped at the expected distance based on library insert size, and (vi) read pairs that mapped in the wrong direction.

## Additional files

Additional file 1: Table S1. List of the individuals and tissue samples in the WGBS datasets analyzed from the NIH Roadmap Epigenomics project. Additional file 2: Table S2. List of the subset of probes from the 450 K that are consistent across tissues of the same individual but that vary between individuals, providing a basis for distinguishing individuals on the basis of methylation in any tissue.

## Abbreviations

450 K: Illumina Infinium HumanMethylation 450 K BeadChip Array
CpG: Cytosine-phosphate-guanine
CpH: Cytosine-phosphate-(non-guanine nucleotide)
DAC: Data Access Committee
EMC: McGill Epigenome Mapping Centre
gDNA: Genomic DNA
GEO: Gene Expression Omnibus
GINA: US Genetic Information Nondiscrimination Act, 2008
IHEC: International Human Epigenome Consortium
NCBI: National Centre for Biotechnology Information
NIH: National Institute of Health
PGP: Personal Genome Project
SNP: Single nucleotide polymorphism
TGCA: The Cancer Genome Atlas
WGBS: Whole-genome bisulfite sequencing
